# Clinical Survival Rate and Laboratory Failure of Dental Veneers: A Narrative Literature Review

**DOI:** 10.3390/jfb15050131

**Published:** 2024-05-16

**Authors:** Tariq F. Alghazzawi

**Affiliations:** 1 Department of Substitutive Dental Sciences, Taibah University, Madinah 42353, Saudi Arabia; tghazzawi@taibahu.edu.sa; 2 Department of Mechanical and Materials Engineering, The University of Alabama at Birmingham, Birmingham, AL 35294, USA

**Keywords:** dental veneer, complication, survival rate, failure, success rate

## Abstract

There is a vast amount of published literature concerning dental veneers; however, the effects of tooth preparation, aging, veneer type, and resin cement type on the failure of dental veneers in laboratory versus clinical scenarios are not clear. The purpose of the present narrative review was to determine the principal factors associated with failures of dental veneers in laboratory tests and to understand how these factors translate into clinical successes/failures. Articles were identified and screened by the lead author in January 2024 using the keywords ‘‘dental veneer”, “complication”, “survival rate”, “failure”, and “success rate” using PubMed/Medline, Scopus, Google Scholar, and Science Direct. The inclusion criteria included articles published between January 1999 and January 2024 on the topics of preparation of a tooth, aging processes of the resin cement and veneer, translucency, thickness, fabrication technique of the veneer; shade, and thickness of the resin cement. The exclusion criteria included articles that discussed marginal and internal fit, microhardness, water sorption, solubility, polishability, occlusal veneers, retention, surface treatments, and wear. The results of the present review indicated that dental veneers generally have a high survival rate (>90% for more than 10 years). The amount of preserved enamel layer plays a paramount role in the survival and success rates of veneers, and glass-ceramic veneers with minimal/no preparation showed the highest survival rates. Fracture was the primary failure mechanism associated with decreased survival rate, followed by debonding and color change. Fractures increased in the presence of parafunctional activities. Fewer endodontic complications were associated with veneer restorations. No difference was observed between the maxillary and mandibular teeth. **Clinical significance:** Fractures can be reduced by evaluation of occlusion immediately after cementation and through the use of high-strength veneer materials, resin cements with low moduli, and thin layers of highly polished veneers. Debonding failures can be reduced with minimal/no preparation, and immediate dentin sealing should be considered when dentin is exposed. Debonding can also be reduced by preventing contamination from blood, saliva, handpiece oil, or fluoride-containing polishing paste; through proper surface treatment (20 s of hydrofluoric acid etching for glass ceramic followed by silane for 60 s); and through use of light-cured polymerization for thin veneers. Long-term color stability may be maintained using resin cements with UDMA-based resin, glass ceramic materials, and light-cure polymerization with thin veneers.

## 1. Introduction

A dental veneer is a layer of material placed on the (front) surface of a tooth to improve appearance and functionality and to protect the tooth surface from damage. There are two main types of materials used to fabricate veneers: composite and dental porcelain. Ceramic veneers are fabricated using computer-assisted design and computer-assisted manufacturing (CAD/CAM) or indirectly fabricated by a technician in a dental laboratory, while composite veneers may be directly placed (built-up in the mouth) or indirectly fabricated in a dental laboratory. Once fabricated, the veneer is adhesively bonded to the tooth, typically using a resin cement. Veneers have gained popularity in esthetic dentistry due to the relative thinness of the restoration, with minimal tooth preparation involved [1,2,3]. The thickness of the veneers is modified to obtain a thinner section of up to 0.3 mm; these veneers are called ultrathin veneers and preserve the structural integrity of the tooth without having to prepare enamel, in contrast to crowns [4]. However, veneer restorations are associated with certain concerns, including the possibility of long-term color changes, because the overall color can be affected by the underlying ceramic, cement, and tooth substrate. The sections of veneers are thinner than those of crowns; therefore, they are more susceptible to fracture [5,6]. Additionally, there is no mechanical mode of retention for laminate veneers; thus, they are more likely to debond [5,6].

There is a vast amount of published literature concerning dental veneers; however, the effects of tooth preparation, aging, veneer type, and resin cement type on the failure of dental veneers in laboratory versus clinical scenarios are not clear. The purpose of the present narrative review was to determine the principal factors associated with failures of dental veneers in laboratory tests and to understand how these factors translate into clinical successes/failures.

## 2. Study Selection

Articles on the survival and failure of veneers were identified using the keywords ‘‘dental veneer”, “complication”, “survival rate”, “failure”, and “success rate” in PubMed/Medline, Scopus, Google Scholar, and Science Direct. The inclusion criteria included articles published between January 1999 and January 2024 on the topics of preparation of a tooth, aging processes of the resin cement, veneer, translucency, thickness, fabrication technique of the veneer, shade, and thickness of the resin cement. The exclusion criteria included articles that addressed marginal and internal fit, microhardness, water sorption, solubility, polishability, occlusal veneers, retention, surface treatments, and wear. These factors were excluded because the aim of this paper is to examine the clinical relevance between the laboratory failures and clinical survival rate and thereby enhance the knowledge of dental practitioners to help decrease failures in their dental practice. 

## 3. Results

Laboratory studies were selected to determine the cause of the failure in the veneers, while clinical studies were selected to determine the important factors that could affect survival and success rates. The types of veneer failures discussed in this paper include fracture, debonding, and color change.

### 3.1. Laboratory Failures

#### 3.1.1. Fracture Failures

**Figure 1:**
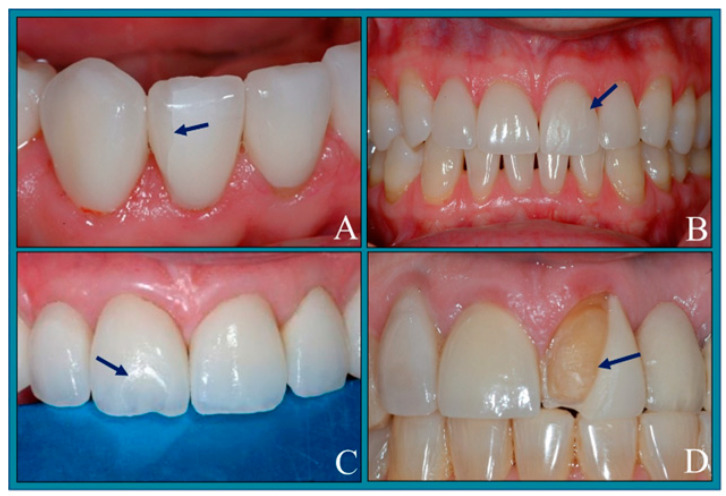
Fracture failure of the laminate veneers. The fracture may start as a crack from the margin and move toward the incisal edge as a result of improper finishing and polishing procedures (**A**,**B**), sudden chipping of the incisal edge as an effect of improper adjustments of the occlusion during centric relation or protrusive movements (**C**), or involvement of the incisal edge with the labial surface (**D**). The photos were sourced from the School of Dentistry, University of Alabama, Birmingham, USA.

Fractures are important complications associated with veneers [7], and the risk of fractures increases with time. The most common location of the ceramic fractures is the incisal edge, as shown in Figure 1. Frequently, fractures occur at the cervical one-third because the enamel is thinner, resulting in dentin exposure that may compromise longevity [8]. The causes of fractures in veneers are summarized in Table 1 [7,9,10,11,12,13,14,15,16,17,18].

##### Die Spacer Thickness

Fracture failures have been correlated with die spacer thickness. Magne et al. [13] reported the correlation between composite thickness and veneer thickness and concluded that most cracked porcelain exhibited a ceramic thickness/composite thickness ratio < 3, resulting in catastrophic failure as the fracture mode. Farag et al. [19] reported that the digital die spacer thickness (20 µm, 40 µm, and 100 µm) did not influence the mean fracture resistance of CAD/CAM-generated lithium disilicate veneers. However, the way that the failure occurred significantly differed at various die spacer thicknesses, and higher digital die spacer settings (20 µm, 40 µm, and 100 µm) were found to decrease the microshear bond strength of lithium disilicate veneers generated by CAD/CAM; thus, 100 µm had the lowest bond strength [20].

##### Stiffness of Supporting Structures

Fracture failures can also be related to the stiffness (modulus of elasticity) of the supporting structures, including the resin cement and the surface tooth layer (dentin vs. enamel) [21]. Substrate stiffness has a significant impact on the fracture of ceramics when the ceramic thickness decreases to 1 mm. A higher modulus of elasticity of the supporting structures correlates to a greater failure load; thus, reducing the fracture loads of ceramics has been shown to be significantly related to the dentin substrate. This potentially results from the lower modulus of elasticity of dentin (18 GPa) compared with that of enamel (70 to 80 GPa). Therefore, a resin with a high modulus of elasticity needs to be selected to increase the failure load of veneers [21]. Furthermore, increasing the thickness of the cement layer can amplify the effect of a low modulus of elasticity of cement (6 to 8 GPa). Therefore, a thinner cement is significantly correlated with greater fracture loads. Therefore, it is recommended to restore the cavity before making the final impression; otherwise, the cement layer becomes thick and negatively affects the fracture resistance. Marcondes et al. [22] reported that bonding ceramic veneers with preheated restorative composite resins (PRCRs) could provide an interface filled with a restorative resin material, leading to optimized mechanical properties.

##### Type of Veneer Ceramic Material

Fracture failures have also been related to the type of ceramic material. When the veneer thickness needs to be increased, such as in cases of lingually tilted teeth, peg lateral cases, and large diastema closure cases, ceramic materials with high flexural strength (glass-ceramic = 450 MPa and zirconia = 1200 MPa) need to be selected. The use of feldspathic porcelain in these cases will result in fracture because of its low flexural strength (60 to 70 MPa) [11].

##### Tooth Preparation

The tooth preparation design also affects veneer fractures. Alghazzawi et al. [11] reported that compared to glass ceramic veneers, feldspathic porcelain veneers failed at lower loads. They also found no significant difference in the failure loads between the window preparation with incisal reduction when compared with the three-quarters veneer preparation design. Jurado et al. [9] reported that complete coverage crowns and veneers with palatal chamfers had the highest fracture resistance values. Single crowns and veneers with palatal chamfer showed no significant difference in fracture strength. Veneers with feather-edge and butt-joint designs provided significantly lower fracture resistance than complete coverage crowns and veneers with a palatal chamfer design. Ustun et al. [23] reported that the incisal bevel preparation design provided a more appropriate geometry for stress distribution than the incisal overlap and feather-edge preparation designs. Lateral forces were found to produce more stress on the tooth and laminate material than vertical forces.

##### Veneer Thickness

Lithium disilicate has a flexural strength of 450 MPa. However, there was no effect of veneer thickness on the failure load following bonding. Mihali et al. [24] reported that no failures were observed in veneers of different thicknesses (0.5 mm compared to those with thicknesses of 1 mm, 1.5 mm, 2 mm, or 2.5 mm) for both prepared and unprepared teeth. De Angelis et al. [25] reported that when lithium disilicate was bonded to dentin, the flexural properties of the entire system improved, and the two different substrates appeared to behave like a single unit compared to those of the conventional cementation procedure. Once adhesively luted, 0.6 mm thick lithium disilicate had the same fracture load and flexural strength as the conventionally luted 1.5 mm thick lithium disilicate. Maunula et al. [26] reported that the failure load of 0.3 mm thick lithium disilicate veneers (2002 ± 427 N) was comparable to that of 0.5 mm veneers. However, Blunck et al. [27] reported that the fracture risk increases with thin veneers and preparations with medium to high dentin portions compared to thicker veneers with preparations in enamel or partially in dentin. Sadighpour et al. [28] reported no significant difference in the failure loads of veneers bonded to intact teeth and to teeth with small class V composite fillings. However, extensive composite fillings were found to compromise the bonding of veneers because of an insufficient enamel structure (less than 40%) [29].

#### 3.1.2. Debonding Failures

Debonding is another important complication documented in the literature; the causes of debonding are summarized in Table 2 [8,13,16,17,28,29,30,31,32,33,34,35,36,37,38,39,40,41]. Debonding is related to the poor bond strength between the internal surface of the veneer and the resin cement, between the prepared tooth and the resin cement layer, or within the resin cement. Adhesive failure between the tooth and resin cement is related to dentin exposure (>50%) [42], the presence of large composite restorations [28], poor surface treatment of the tooth, and contamination [40]. 

##### Veneer Surface Treatment

The adhesive failure between the ceramic and resin cement is caused by poor surface treatment of the veneer internal surface and contamination [40]. Zirconia veneers fail mainly due to debonding [11]; this occurs when the adhesive between the zirconia and resin cement fails because the zirconia is purely polycrystalline, with no glass. As a result, zirconia cannot be etched with hydrofluoric acid (HF); HF etching is performed on glass ceramics. Glass-ceramic and feldspathic porcelain veneers fail mainly due to fracture [11] and to a lesser degree due to debonding [11]. Glass-ceramic debonding is more closely related to improper hydrofluoric acid etching (e.max = 20 s, feldspathic porcelain = 60 to 90 s); here, washing and drying are performed and then silane is applied (silane = 60 s) and dried. Martins et al. [38] reported that hydrofluoric acid followed by silanization is the most suitable surface treatment for the cementation of lithium disilicate glass ceramics. Alammar et al. [43] stated that airborne particle abrasion and special phosphate monomer-containing primers or composite resin cements can provide long-term durable resin bonds. Glass-ceramic veneers are recommended to be etched for less than 20 s for lithium disilicate and between 60 to 90 s for feldspathic porcelain.

##### Tooth Preparation

Debonding can be caused by overpreparing the tooth and dentin exposure (>50%), and the degree of dentin exposure has no effect on the survival rate [34]. However, immediate dentin sealing (IDS) has been used to improve the bond strength of dentin to resin-based restorations regardless of the adhesive strategy used [30,39]. Zhu et al. [29] reported that the shear bond strength of the veneers bonded to 100% enamel on finishing surfaces (nearly 20 MPa) was twice that of veneers bonded to 0% enamel (nearly 10 MPa). No significant difference was observed among the 40–100% enamel groups, while the 20% and 0% enamel groups demonstrated a significantly lower mean shear bond strength than the 40% enamel group.

##### Tooth Contamination

**Figure 2:**
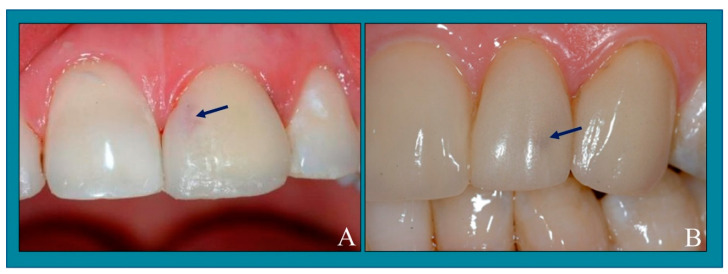
Veneer contaminated with blood during cementation (**A**,**B**). If the contamination is low, it will appear late. However, if the contamination is high, it will immediately occur. A thinner veneer correlates to an increased chance of blood contamination. In this case, the solution was used to cut the veneer, and it was replaced with a new veneer. The bleeding needed to be controlled using astringent solutions, such as aluminum chloride. The photos were sourced from the School of Dentistry, University of Alabama, Birmingham, AL, USA.

Contamination of the tooth with sulcular fluids, blood, or saliva before cementation is also an important factor in debonding failure [40]. Failure frequently occurs from bleeding due to gingivitis resulting from poor provisional veneers (thick margins, rough surface) [33], a forgotten retraction cord from a previous impression, or residual resin cement from provisional veneers. It can manifest as red spots on the facial surface of the veneer (Figure 2). With failed veneers, part of the enamel needs to be removed to eliminate the previous resin cement to expose the dentin. Several authors have suggested using an erbium, chromium: yttrium-scandium-gallium-garnet (Er,Cr:YSGG) laser to safely remove the veneer without damaging the area underneath the enamel layer [34].

#### 3.1.3. Color Failures

The causes of color failures are summarized in Table 3 [16,17,30,41,44,45,46,47,48,49,50,51]. Color changes can be caused by the veneer thickness, material type, substrate color, and ceramic color [16,17,41]. 

##### Literature Concerning Color Changes

Mekled et al. [16] reported that the thickness and background shade need to be considered when selecting zirconia veneers to ensure optimal color matching and overall aesthetic outcomes. Khosravani et al. [17] reported that the color change was greater for thinner ceramics. Different shades of resin cement and layers of ultratranslucent multilayered zirconia veneers affected the final color. Sen et al. [52] stated that the restorative material type, substrate color, and resin cement shade affected the masking ability of monolithic CAD-CAM veneers. Furthermore, Alghazzawi et al. [53] reported that zirconia specimens exhibited greater color changes with thermal aging than glass ceramics.

### 3.2. Clinical Failures

Clinical failure can be defined in terms of the survival and success rates of the veneers in the oral environment; this failure can be affected more than the other factors (fracture, debonding, color change) at the same time. Survival and success rates for veneers have been extensively reported in the literature, as shown in Table 4 [5,7,24,30,32,42,54,55,56,57,58,59,60,61,62,63,64]. Different factors need to be evaluated during the maintenance phase to evaluate the longevity of the restoration. Moreover, veneers bonded to enamel were found to be substantially stronger and more damage tolerant than those bonded to dentin or half enamel/half dentin [29]. The amount of exposed dentin has been discussed as a risk factor for the clinical failure of the ceramic veneer restorations [42].

Cemented veneers tend to change color over time, which is often referred to as color aging. Triethylene glycol dimethacrylate (TEGDMA)-based resins can release higher quantities of monomers into aqueous environments than bisphenol A-glycidyl methacrylate (Bis-GMA)- and urethane dimethacrylate (UDMA)-based materials and cause discoloration over time. The yellowing of a material over time could be related to an increased amount of camphorquinone (CQ) in its formulation. Additionally, larger particle sizes and higher particle counts were more susceptible to discoloration despite the presence of UDMA and Bis-BMA monomers [65]. The discoloration can be yellow or red [48]. The natural process of aging is responsible for the darkening of teeth, and this also affects the long-term color of the veneers. Compared to darker shades, lighter shades are more affected by color change over time. The use of dual-cure cement for veneers less than 1 mm in thickness often results in yellowish discoloration over time. Castellanos et al. [66] reported that amine-free cements containing Ivocerin (IVO) + diphenyl(2,4,6-trimethylbenzoyl)-phosphine oxide (TPO) are a better alternative to camphorquinone and amine (CQ-amine) cements with regard to yellowing color changes. Favarão et al. [67] reported that CQ/ethyl 4-(dimethylamino) benzoate (EDMAB) + TPO exhibited the greatest color stability. Delgado et al. [68] reported that Ivocerin alone or +TPO was an effective alternative photoinitiator to substitute for CQ. The resin cement containing only TPO had lower bond strength values than the resin cements with CQ, Ivocerin, and Ivocerin + TPO. Kavut et al. [50] reported that light-cure resin cements are preferable for full ceramic restorations because of their long-term color stability.

## 4. Discussion

In this narrative review, the most prevalent causes of laboratory (*in vitro* studies) failures of veneers were revealed, and the factors that affected survival and success rates and the causes of reduced survival rates were determined. Laboratory studies have the advantage of being more rapid and providing immediate results. However, laboratory studies generally do not use extracted teeth because of variations in size, shape, time of extraction, and storage with uncontrolled tooth preparation. Overall, artificial tooth substrates are more standardized and controlled. Therefore, laboratory studies use resin tooth substrates to simulate the modulus of elasticity of dentin (18 GPa) [21] but generally do not replicate the exact clinical loading substrate. Long-term clinical trial studies (*in vivo* studies) are needed to confirm the laboratory causes of failure. Clinical studies have the disadvantage of being time-consuming.

The amount of preserved enamel layer plays an important role in the survival and success rates [5] of dental veneers, and the minimal/no preparation veneers have the highest survival rate [5] because there is no dentin exposure and the bonding is intact within the enamel layer. Additionally, glass-ceramic veneers have a higher survival rate than the feldspathic veneers because feldspathic porcelain has a higher glass concentration and is more liable to experience color changes; in contrast, the glass ceramic is more crystalline, increasing the fracture strength. Additionally, teeth that have minimal/no preparations have fewer endodontic complications [30,42], but this can affect long-term color changes. No difference in the survival rate was observed between maxillary and mandibular teeth, even though the enamel layer is thinner in mandibular anterior teeth in addition to the occlusion [42]. No difference was found in the survival rate between males and females.

Notably, it was seen from the clinical studies that the most prevalent failure for veneers is fracture [5,24,54,61] because the veneer restoration is thin (0.3–0.5 mm) and more liable to fracture; preventing this postoperative failure is dependent on good occlusion in centric-related, protrusive, and lateral excursions immediately after veneer cementation [14]. Fracture failures can be amplified by the presence of parafunctional activities [7]; therefore, a night guard is recommended for patients. Dentists need to examine occlusions carefully, especially for any parafunctional habits such as bruxism, before initiating treatment and after cementation of the veneers because an unfavorable occlusion is the leading cause of fracture failure [9].

Debonding is considered the second most common failure mode after fracture because of the lack of mechanical retention from tooth preparation for the veneers; retention is promoted by chemical adhesion with the resin cement. The size of the preexisting composite resin restorations on the teeth to be veneered is a determining factor for debonding failures; a smaller restoration results in greater preservation of the enamel layer and thus low debonding failure [28,29], and a large preexisting restoration results in greater failure because a smaller amount of the enamel layer is preserved. Furthermore, the process of erosion (loss of enamel) promotes debonding.

The dentist can help to increase the survival rate of the veneers by reducing clinical failure; fractures can be reduced with occlusion evaluation immediately after cementation, using high flexural strength veneer materials for bruxer patients and selecting resin cements with low moduli and thin layers for highly polished veneers. Additionally, debonding failures can be reduced with minimal/no preparation, and IDS should be considered when the exposed dentin is >50%; debonding can also be reduced by the following: contamination prevention from blood, saliva, handpiece oil, fluoride containing polishing paste; proper surface treatment (20 s of hydrofluoric acid etching for glass ceramics and 60–90 s for feldspathic porcelain followed by silane for 60 s); and use of light-cured polymerization for thin veneers (<1 mm). Long-term color stability can be maintained using a resin cement with UDMA-based resin, a smaller filler size, a lower filler content, a veneer material with glass ceramic material over feldspathic porcelain, light-cured polymerization for thin veneers, and internal bleaching during previous endodontic treatment.

When the substrate shade is light, a low translucency glass ceramic, medium translucency glass ceramic, or feldspathic porcelain can be selected by the dental laboratory. The thickness of the veneer needs to be less than 1 mm, and the dental practitioner can use translucent resin cement to allow the use of light-cured resin cement. However, if dark substrate is involved, good communication needs to occur between the dental laboratory and the dental practitioner, and ceramic materials with medium opacity, high opacity glass-ceramics, or zirconia should be selected. The dental practitioner may request that the dental laboratory supplies a thicker veneer (>1 mm) to mask the dark underlying tooth structure, and an opaque resin cement or possibly a dual-cured resin cement can be used; otherwise, immediate color failure will be evident after cementation [16,17].

In summary, dentists should use veneers as the first choice for esthetic restoration, if possible, because the required tooth preparation is minimal/no preparation is necessary, and thus the enamel layer is preserved, increasing the survival rate. The survival rate of dental veneers could be increased by limiting the factors affecting fracture, debonding, and color change. Long-term clinical studies using ultratranslucent zirconia veneers are needed, especially for patients with parafunctional activities and dark substrates.

## 5. Conclusions

Dental veneers generally have a high survival rate (>90% for more than 10 years). The survival rate can be optimized with minimal/no tooth preparation and by using glass-ceramic veneers. Fracture is considered the primary factor affecting the clinical survival rate, followed by debonding. While laboratory studies may reproduce clinical failures, long-term clinical studies are necessary to accurately predict the survival of veneers.

## Figures and Tables

**Table 1:** The main factors affecting veneer fractures, with examples.

Factor	Examples
Inappropriate case selection	Unfavorable occlusion [9].
	Endodontically treated teeth [7].
	Patient with inherent parafunctionality, e.g., grinding (ice cubes), biting (nail and pencil), bruxism [7].
Improper material selection	Selection of resin cements with a low modulus of elasticity [10].
	Selection of a material with a low flexural strength (feldspathic porcelain) for cases that need a high strength, e.g., lingually tilted teeth, diastema closure, and/or correction of malformed anterior teeth [11].
Improper communication with the dental laboratory	Thicker veneer [12] and incorrect ratio of veneer thicknesses to die spacer (the die spacer thickness must not be more than 1/3 of the veneer thickness to prevent debonding or fracture) [13].
Improper preparation design	Sharp angles or inadequate tooth reduction; extension of the preparation to the palatal surface [9]; incisal coverage for maxillary canine [14]; not restoring a cavity to obtain a thick cement layer [12]; labial thicknesses of ultrathin veneers should be 0.5/0.4 mm for premolar teeth [15].
Improper cementation procedure	Improper veneer handling, especially for fragile feldspathic veneers.
	Incomplete polymerization using light-cure based resin cements for thick (>1 mm) opaque cement and opaque veneer (e.max MO *, HO *, zirconia) [16,17].
	Inappropriate finishing and polishing, leading to cracks [18].
Improper occlusion for post laminate veneer delivery	Inappropriate occlusion in centric relation, protrusive, and canine guided movements [14].

* MO = medium opacity, HO = high opacity.

**Table 2:** The main factors affecting veneer debonding, with examples.

Factor	Examples
Inappropriate case selection	The patient has poor oral hygiene and gingivitis, resulting in bleeding during cementation [30,31].
	Presence of large preexisting composite resin; insufficient enamel layer for bonding [28,29].
	Severe erosion; completely dissolved enamel layer [32].
Improper diagnosis and treatment planning	Preparation of the enamel is not needed, e.g., lingually tilted tooth [8,29,32].
Improper provisional veneers	Thick margins and rough surfaces promote food collection, leading to bleeding during cementation [33]. Additionally, residual cord fragments from impressions and residual resin cement promotes bleeding during cementation
Previous debonded veneer	Old veneers were removed mechanically, exposing the dentin, without using laser technology [34].
Improper communication with the dental laboratory	Incorrect ratio of veneer thicknesses to die spacer (die spacer thickness must not be more than 1/3 the veneer thickness to prevent debonding or fracture) [13].
	Etching the veneer with hydrofluoric acid without the knowledge of the dentist [35].
Improper material selection	Use of polishing paste containing fluoride [36] or oil [37].
	Silane coupling agent used is not fresh [38].
Over-tooth preparation	Exposed dentin of ≥50%; IDS * is not used [30,39].
Improper isolation and tissue management	The sulcular fluids can be controlled with retraction cords. Saliva can be controlled with lip retractors. Bleeding can be controlled by astringents (aluminum chloride) [40].
Poor cementation techniques	Hydrofluoric acid is not used properly (the veneer is etched twice, is over-etched, or is not etched at all) [35].
	Contamination of the veneer after hydrofluoric acid etching and/or silane application; moisture/oil contamination from air syringe [37].
	Incomplete polymerization using light-cure based resin cements for thick (>1 mm) opaque cement and opaque veneer (e.max MO *, HO *, zirconia) [16,17,41].

* IDS = immediate dentin sealing, MO = medium opacity, HO = high opacity.

**Table 3:** The main factors affecting veneer color changes, with examples.

Factor	Examples
Improper patient selection	The patient is a heavy smoker (marginal discoloration) [30] and has poor oral hygiene.
	The tooth underwent a previous endodontic treatment [30].
Improper material selection	Selection of a material (feldspathic porcelain) for cases that need staining or are adjacent to crowns [16].
Improper communication with dental laboratory	Failure to select the opacity (MO *, HO *, zirconia) of dark teeth occurring after tooth preparation due to poor communication with the dental laboratory [16,17,41,44].
	Making a thinner veneer for a dark substrate [16,17,41,44].
	Producing a thick veneer without an appropriate reason (thicker veneers decrease the translucency) [16,17,41,44].
	Large number of firing cycles are used, which will burn the coloring metallic oxides and the veneer will be darker [45]; the quantity and position of the veneers during firing [46].
	Poor glazing and polishing [47].
Normal aging process of the tooth	The tooth has ability to change color over time [48].
Improper cementation technique	No verification of the veneer color occurred before cementation by using try-paste [49].
	Use of dual-cure resin cement for thin veneers (≤1 mm), with HT *, LT *, and MT * glass-ceramics [41,50]
Microleakage presented as a dark line at the gingival margin	Lack of bonding agent; use of a scaler to remove resin cement; subgingival margin at the dentin or root surface is more likely to be prone to leakage, poor isolation, and tissue management (proper subgingival margin isolation before and during bonding is vital to prevent interference from the sulcular fluids with the bonding surfaces, which causes yellowish discoloration.); use of thick adhesive layer; and lack of margin fit [51].

* TEGDMA = triethylene glycol dimethacrylate, CQ = camphorquinone, TPO = diphenyl(2,4,6-trimethylbenzoyl)-phosphine oxide, MO = medium opacity, HO = high opacity, HT = high translucency, LT = low translucency, and MT = medium translucency.

**Table 4:** The main reasons for the failures and survival rates of veneers.

Study	Failure Cause	Preparation Type	Survival/Success Rate and Material
De Angelis et al. [54] 2023	Five relative failures (3 minimal fractures or chips and 2 limited marginal discolorations) and 2 absolute failures (unrepairable fractures)	No-prep porcelain laminate veneers	The mean observation period was 43.1 months, with an observation interval of 36 to 60 months, a survival rate of 97.4%, and a success rate of 91.0%.
Limet et al. [55] 2023	Surface roughness, color mismatch, and marginal discoloration	NONE	The overall pooled survival rate of the randomized controlled trials was 88% (95% CI *: 81–94%), with the mean follow-up time ranging from 24 to 97 months.
Yıldırım et al. [56] 2023	Small marginal fractures	NONE	It was found that 73% (n * = 22) of the PLVs * had perfect marginal adaptation, and 57% (n * = 17) of the PLVs were evaluated as a good color match (no difference in shade and/or translucency).
Sen et al. [57] 2023	NONE	NONE	According to the ceramic system used, the estimated Kaplan–Meier survival rate was 92.7% for Emax-CAD * and 89.1% for feldspathic ceramic. Survival rates were significantly affected by the location of the veneer.
Kam Hepdeniz et al. [58] 2023	Four debonding (marginal adaptation, score 4) and 3 fractures (fracture of restoration, score 3)	No tooth preparation	The overall survival rate was 91.3% after 7 years.
Silva et al. [59] 2023	No absolute failures such as debonding, veneer fracture, or secondary caries. Superficial marginal discoloration was observed in one element (maxillary left lateral incisor) of one patient	NONE	After a mean follow-up of 4.33 years (4–5 years), a survival rate of 100% was detected for the 28 minimally invasive ultratranslucent zirconia veneers cemented in the 3 patients.
Mihali et al. [24] 2022	In this retrospective survival analysis, the failures, including the fracture of veneers and dental hard tissue, occurred both in prep and no-prep teeth. No failures were observed in veneers with a maximum thickness of 0.5 mm compared to those with a maximum thickness of 1 mm, 1.5 mm, 2 mm, and 2.5 mm	Prep and no-prep	The overall survival rate was 91.77% for up to 7 years of function, with a failure rate of 8.23%.
Tekçe et al. [60] 2022	Fracture (12.4%)	Prep for amelogenesis imperfecta	Survival rate: 80.5% after 4 years for nanohybrid and 92.5% for nanofill composite.
Smielak et al. [5] 2022	Eight restoration chipping/fractures, one debonding, and one fracturing of the tooth	Conventional prep and no-prep/minimally prep	Survival rate: 9.67% for conventional veneers and 100% for no-prep/minimal prep veneers Mean success rate time for conventional veneers without absolute or relative failures was 9.32 years, and 10.28 years for no-prep/minimally invasive veneers.
Demirekin et al. [61] 2022	Fracture and marginal discoloration	Incisal edge and part of the palatal/lingual side of the tooth	Survival rate: 99.7% after 10 years for IPS e.max Press.
Mazzetti et al. [62] 2022	Composite veneers presented a higher risk of failure than ceramic veneers with higher HR * for survival [HR 4.00 (2.74–5.83)] and success [HR 5.16 (2.65–10.04)]	NONE	Considering success analysis, AFR * for veneers in 5 and 10 years were 9.1% and 10% for direct composite and 2.9% and 2.8% for ceramic, respectively. Survival analysis showed an AFR * of 3.9% and 4.1% for composite and 1.4% and 1.2% for ceramic over the same periods.
Fotiadou et al. [7] 2021	Fracture, debonding, endodontic complications, and recurrence of caries	NONE	Survival and success rates of lithium disilicate indirect restorations were calculated at 6.6 years to be 96.3% and 93.8%, respectively. After 8.5 years, the survival rate was calculated at 94% and the success rate at 83.8%.
Gonzalez-Martin et al. [32] 2021	Total fracture occurrence was 9.8% in 13 participants. No fractures were observed in prep veneers, while 16 out of 125 min-prep and 3 out of 57 no-prep veneers had fractures	Twelve veneers were prep, 125 were min-prep, and 57 were no-prep.	A generalized estimating equation model revealed that the OR * of veneer fracture was significantly higher in men (OR = 11.29), in patients who exhibited tooth wear at baseline (OR * = 5.54), and in central (OR * = 13.56) and lateral (OR * = 10.43) incisors compared to canines and premolars.
Rinke et al. [42] 2020	Nine re-cementations, two endodontic treatments, two composite fillings, and one fracture polishing. The jaw position (maxilla/mandible, survival *p* = 0.578/success *p* = 0.056) had no influence on the clinical performance	NONE	The 10-year survival rate was 91.8% [95% CI *: 0.87;0.97]. Seventy-seven of the 101 restorations remained intervention-free in service (success rate: 78.6% [95% CI *: 0.70;0.88]).
Aslan et al. [63] 2019	Failures were seen in 1.64% of the restorations (fractures and debonding in 0.55% and 1.09%, respectively)	NONE	Survival rate: 97.4% after 10 years.
Gresnig et al. [30] 2019	Nineteen failures were observed in the form of debonding (n * = 3), fracture (n * = 15), and extraction due to endodontic complications (n * = 1)	NONE	Teeth with more than 50% dentin exposure significantly benefited from IDS *. Preexisting restorations or endodontic treatments did not have an effect on the survival rate of ceramic laminate veneers. However, smoking habits and previous endodontic treatments negatively affected the success rate due to color changes.
Morimoto et al. [64] 2016	Debonding: 2% (95% CI *: 1% to 4%); fracture/chipping: 4% (95% CI *: 3% to 6%); secondary caries: 1% (95% CI *: 0% to 3%); severe marginal discoloration: 2% (95% CI *: 1% to 10%); endodontic problems: 2% (95% CI *: 1% to 3%); and incisal coverage (OR *: 1.25) (95% CI *: 0.33 to 4.73)	NONE	The estimated overall cumulative survival rate was 89% (95% CI *: 84% to 94%) for a median follow-up period of 9 years. The estimated survival for glass-ceramic was 94% (95% CI *: 87% to 100%), and for feldspathic porcelain veneers, 87% (95% CI *: 82% to 93%).

* CAD = computer-assisted design, PLVs = porcelain laminate veneers, CI = confidence interval, n = number of specimens, HR = hazard ratio, AFR = annual failure rate, OR = odds ratio, and IDS = immediate dentin sealing.
